# Phosphate-Solubilizing Bacteria: From Phosphate Mobilization to Improved Plant Nutrition

**DOI:** 10.3390/microorganisms14102301

**Published:** 2026-10-09

**Authors:** Dilfuza Egamberdieva, Narges Samanian, Durdigul Juraeva, Shahlo Satimova, Ulugbek Sadullayev, Laziz Joniqulov

**Affiliations:** 1 International Center for Strategic Development and Research, Ministry of Agriculture of the Republic of Uzbekistan, Tashkent 100140, Uzbekistan; n.samanian@gmail.com (N.S.); durdiguljuraeva@gmail.com (D.J.); sadulaevun@gmail.com (U.S.); janikulovlaziz@gmail.com (L.J.); 2 Faculty of Biology, National University of Uzbekistan, Tashkent 100174, Uzbekistan; shahlosatimova93@gmail.com; 3 Department of Functional Food Technology, Tashkent Institute of Chemical Technology, Tashkent 100011, Uzbekistan

**Keywords:** microbial phosphorus cycling, phosphorus use efficiency, plant nutrition, biofertilizers, sustainable nutrient management

## Abstract

Phosphorus (P) is a vital macronutrient required for plant growth and development; however, only a small fraction is readily available for plant uptake because much of the soil phosphorus becomes immobilized through chemical fixation. Moreover, dependence on finite phosphate rock reserves and the intensive use of mineral phosphorus fertilizers increase production costs and raise environmental concerns, emphasizing the need for more sustainable phosphorus management strategies. Phosphate-solubilizing bacteria (PSB) are key drivers of microbial phosphorus cycling, enhancing phosphorus bioavailability by transforming insoluble inorganic and organic phosphorus compounds into forms that plants can readily absorb. This process is mediated by the secretion of organic acids, phosphatases, phytases, and other metabolites involved in phosphorus mobilization. In addition to increasing phosphorus bioavailability, PSB can enhance overall nutrient-use efficiency, promote root growth and architecture, support biological nitrogen fixation, and strengthen plant resilience to abiotic stresses, including drought, salinity, and temperature extremes. Owing to these multiple plant growth-promoting functions, PSB are increasingly recognized as promising bioinoculants that can improve crop productivity, enhance climate resilience, and reduce reliance on synthetic phosphorus fertilizers. This narrative review provides a comprehensive overview of recent advances in understanding PSB-mediated microbial phosphorus cycling, with particular emphasis on the mechanisms underlying phosphorus solubilization, interactions with plant roots, and contributions to sustainable crop production. Evidence from laboratory, greenhouse, and field studies is synthesized to evaluate PSB-mediated mechanisms, plant responses, and their potential for agricultural application. The review also highlights key barriers to the widespread adoption of PSB, including inconsistent field performance and challenges associated with commercialization. Addressing these limitations could support the development of cost-effective biofertilizers that increase phosphorus-use efficiency, reduce dependence on mineral fertilizers, lower production costs, and enhance farm profitability while contributing to sustainable agriculture.

## 1. Introduction

Phosphorus (P) is an essential macronutrient required for virtually all aspects of plant growth and development. It is a structural component of nucleic acids and phospholipids and plays essential roles in energy transfer through adenosine triphosphate (ATP), photosynthesis, respiration, signal transduction, and membrane integrity. These functions underpin fundamental physiological processes, including cell division, root development, biomass accumulation, and reproductive growth in plants [1,2,3].

Soil phosphorus (P) originates from natural sources, mainly the weathering of P-bearing minerals and organic matter such as plant residues and animal inputs. Agricultural practices further increase P inputs through mineral fertilizers, manure, compost, and other amendments. However, only a small fraction of total soil P is readily available to plants, primarily as soluble orthophosphate (H_2_PO_4_^−^ and HPO_4_^2−^). Much of the remaining P is present in insoluble mineral forms, strongly bound to Fe and Al oxides in acidic soils, or precipitated with Ca in alkaline soils, resulting in low P availability despite substantial total soil P. Consequently, the disparity between total soil phosphorus reserves and plant-available phosphorus remains a major constraint on agricultural productivity and phosphorus use efficiency worldwide [4,5,6]. Thus, phosphorus availability is determined not only by total soil phosphorus content but also by its chemical forms and the dynamic processes regulating P transformation in the soil environment [7,8]. To compensate for low phosphorus availability, large quantities of phosphate fertilizers have been applied worldwide. However, only a limited proportion of applied phosphorus is typically utilized by crops during the growing season, while the remainder becomes immobilized in soil or is gradually lost through runoff and erosion, contributing to environmental degradation and reducing long-term phosphorus-use efficiency [5,9].

**Figure 1:**
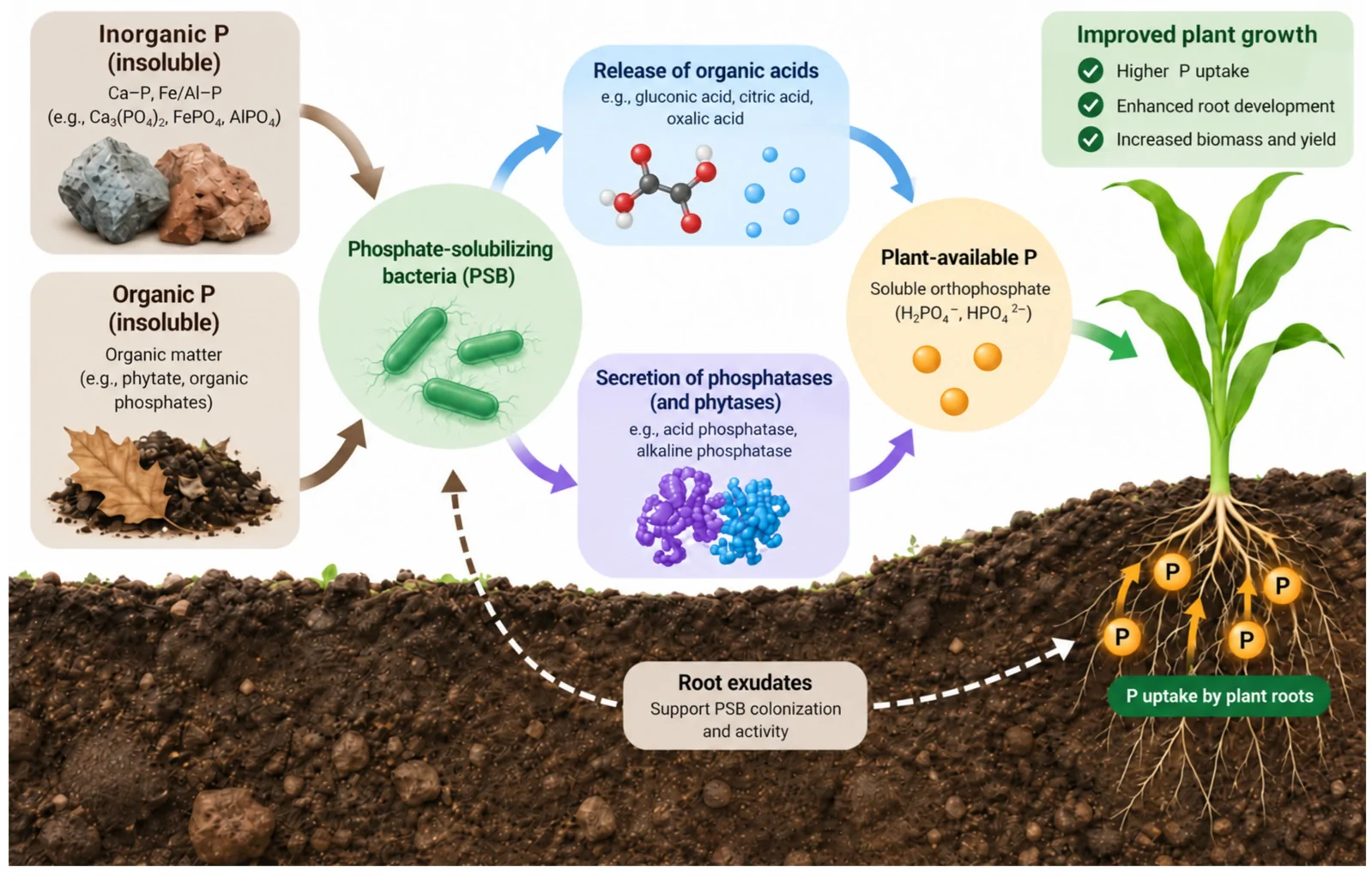
Schematic representation of PSB-mediated solubilization and mineralization of soil phosphorus, increasing plant-available phosphorus and its subsequent uptake by plant roots to support plant growth.

In recent decades, increasing attention has been directed towards harnessing beneficial soil microorganisms to improve phosphorus availability and enhance phosphorus-use efficiency in agricultural systems [4,10,11]. Among these microorganisms, PSB constitute a functionally diverse group capable of converting insoluble inorganic and organic phosphorus compounds into plant-available forms through a range of biochemical processes. This process is primarily mediated through the secretion of organic acids, such as gluconic, citric, oxalic, and malic acids, which lower the pH in the rhizosphere and promote the dissolution of mineral phosphates. PSB can also release phosphatases and phytases that hydrolyze organic phosphorus compounds, further increasing the pool of plant-available phosphorus. These complementary pathways of PSB-mediated phosphorus mobilization and their relationship to plant phosphorus uptake are illustrated in Figure 1. By increasing phosphorus availability in the rhizosphere, PSB can enhance phosphorus acquisition by plants, improve crop growth, and reduce dependence on chemical phosphorus fertilizers, making them promising components of sustainable nutrient management strategies [12,13,14]. Beyond phosphorus mobilization, many PSB interact closely with plant roots and the surrounding microbial community, where their contribution to plant performance may extend beyond nutrient acquisition [4,15]. Recent advances in rhizosphere microbiology have highlighted that the effectiveness of PSB is influenced by complex interactions among microbial communities, plant hosts, and environmental conditions, emphasizing the importance of understanding their ecological functions in addition to their P-solubilizing capacity [16].

Despite substantial advances in the characterization and application of PSB, several challenges continue to limit their effective utilization in agricultural systems. Most studies have focused on the isolation of efficient strains and the evaluation of their plant growth-promoting potential under controlled conditions, whereas comparatively less attention has been given to integrating bacterial diversity, phosphorus-solubilization mechanisms, plant–microbe interactions, and environmental factors that collectively determine PSB performance under field conditions [17]. Recent reviews have summarized specific aspects of PSB, including their diversity, mechanisms of phosphorus mobilization, and agricultural applications [14,18,19]. However, rapid advances in rhizosphere microbiology, microbial ecology, and multi-omics approaches, together with increasing interest in sustainable nutrient management, warrant an updated synthesis that integrates these developments into a unified framework for understanding the role of PSB in sustainable agriculture [8,15].

This review provides a comprehensive synthesis of current knowledge on PSB and their role in sustainable agriculture. It examines the taxonomic diversity of PSB, the biochemical and molecular mechanisms underlying phosphorus mobilization, and their interactions with plants and the rhizosphere microbiome. In addition, the review discusses the environmental and edaphic factors influencing PSB performance, recent advances in PSB-based biofertilizers, the challenges of their large-scale implementation, and the economic feasibility of PSB adoption, including cost-effectiveness, reduced fertilizer inputs, improved phosphorus-use efficiency, and enhanced farm profitability.

Specifically, this review addresses how PSB diversity, phosphorus-mobilization mechanisms, plant–microbe interactions, and environmental conditions collectively determine agronomic performance and how this knowledge can be translated into reliable and economically viable PSB-based biofertilizers. Unlike reviews that primarily address individual aspects of PSB biology or application, this review integrates mechanistic and ecological understanding with evidence of field performance, climate resilience, and economic and commercialization constraints. Economic evidence is evaluated using reported quantitative outcomes, including fertilizer savings, yield response, net return, and benefit–cost ratio, while distinguishing between PSB supplementation of conventional fertilization and PSB-assisted partial substitution of mineral phosphorus.

## 2. Ecological Distribution of Phosphate-Solubilizing Bacteria

PSB are widely distributed across diverse terrestrial ecosystems, including agricultural and natural soils, plant rhizospheres, endophytic tissues, composts, and organic amendments, as well as environments exposed to salinity, drought, and nutrient limitation [6,14,20]. Agricultural soils represent important reservoirs of PSB, where their communities are shaped by crop cultivation, fertilization, and soil physicochemical conditions [21,22]. Factors such as soil pH, P availability, organic matter, moisture, vegetation, and land-use practices influence PSB abundance, diversity, and distribution [12,15,23]. Plants also affect PSB communities through root exudates, which modify the surrounding soil environment and promote selective recruitment. Consequently, PSB populations in rhizosphere and endophytic niches may differ substantially from those in bulk soil, reflecting host–microbe interactions and adaptation to specific ecological conditions [16].

Habitat-specific adaptation affects bacterial survival, persistence, colonization, and the consistency of P mobilization under field conditions, making ecological context important for PSB-based biofertilizer development [6,10,24]. Soil properties, particularly pH, P availability, organic matter, and structure, strongly influence PSB distribution [25]. PSB may also be unevenly distributed among soil aggregate fractions, highlighting the role of soil microhabitats [22]. Differences in salinity and alkalinity further influence the taxonomic composition and P-solubilizing efficiency of native PSB communities, indicating that environmental filtering shapes their distribution and function [21,26]. Agronomic management influences PSB communities by modifying soil nutrient status and microbial habitats. Restoration of degraded agricultural land can promote PSB establishment and enhance microbial P cycling, demonstrating the effects of land-use practices on PSB diversity and function [27]. Similarly, PSB application in combination with P fertilizers can alter bacterial community composition and soil P fractions in cultivated Phaeozem soils, highlighting the interactions between nutrient management and soil microbial ecology [28]. Cultivated soils are also important sources of indigenous PSB with agricultural potential [29,30,31].

In bulk soil, PSB occur within the broader microbial community and are shaped primarily by soil driven environmental filtering. Their abundance, diversity, and activity vary with pH, texture, organic matter, moisture, salinity, mineral composition, and phosphorus status, which influence both community structure and the expression of phosphate solubilizing functions [32]. The functional significance of PSB is therefore context-dependent and closely linked to the chemical heterogeneity of soil P pools. While PSB can mobilize mineral-associated and organic P through diverse biochemical processes, the released phosphate may subsequently be immobilized by microbial biomass or re-associated with Fe, Al, and Ca minerals. Thus, PSB mediated P mobilization in bulk soil depends not only on microbial abundance and solubilization capacity but also on the retention, transformation, and subsequent fate of mobilized P [33]. Importantly, bulk soil should not be considered simply as a source of PSB inoculum. It represents an ecological reservoir in which PSB populations are maintained, selected, and functionally constrained by the soil environment. Only a fraction of this community may subsequently become enriched near plant roots, where root-derived carbon and other signals create a markedly different ecological niche. This transition from a soil-filtered PSB community to a plant-influenced rhizosphere community provides the basis for understanding how environmental occurrence is translated into plant-associated function.

The rhizosphere is one of the most dynamic microbial habitats in terrestrial ecosystems and represents the primary ecological niche for PSB. Continuous release of root exudates, including sugars, amino acids, organic acids, phenolics, and secondary metabolites, creates nutrient rich microenvironments that support dense and metabolically active microbial communities [34,35]. The composition of these exudates is strongly influenced by plant nutritional status, particularly phosphorus availability, which in turn shapes rhizosphere microbial community structure. Comparative analyses of teosinte and modern maize demonstrated that phosphorus availability significantly altered root exudate profiles and was associated with changes in rhizosphere microbial community composition, indicating that both nutrient status and host genotype contribute to microbial community assembly [36]. Complementing these findings, Pantigoso et al. [37] demonstrated that several metabolites enriched in phosphorus-deficient root exudates enhanced P-solubilizing activity in bacterial strains without consistently stimulating bacterial growth, suggesting that plants can regulate microbial phosphorus-mobilizing functions through specific biochemical signals rather than solely by increasing carbon availability. Similar observations were reported for rhizosphere bacteria associated with cover legumes cultivated in acidic agricultural soils, where numerous indigenous isolates were capable of solubilizing multiple inorganic phosphate sources, demonstrating adaptation to chemically restrictive environments [30]. Together, these studies indicate that the rhizosphere harbors functionally diverse PSB communities whose composition reflects interactions among host plant species, soil properties, and local environmental conditions rather than a conserved taxonomic assemblage.

Successful colonization of the rhizosphere is a prerequisite for sustained plant–microbe interactions and effective phosphorus mobilization. Using a green fluorescent protein (GFP)-labeled PSB isolated from rhizosphere soil, Wang et al. [38] demonstrated stable colonization of Chinese cabbage roots throughout plant development, with bacterial cells initially colonizing the root surface before progressively occupying internal tissues. These observations illustrate that certain rhizosphere-derived PSB possess strong rhizosphere competence and can maintain persistent associations with host plants. Beyond physical colonization, PSB may also influence the broader microbial environment surrounding roots. In wheat, inoculation with PSB increased phosphorus availability and modified microbial interactions in both the rhizosphere and endosphere, supporting the concept that phosphorus mobilization by PSB can indirectly influence the organization and functional potential of plant-associated microbial communities [39]. Likewise, phosphorus deficiency significantly modified the diversity, taxonomic composition, and co-occurrence networks of endophytic bacterial communities in rice roots, demonstrating that phosphorus stress affects both community structure and microbial interactions within the endosphere [40]. Oteino et al. [41] isolated endophytic *Pseudomonas* strains with P-solubilizing ability that also produced indole-3-acetic acid and siderophores and promoted plant growth under experimental conditions.

These findings indicate that some endophytic PSB possess multiple functional traits that may contribute simultaneously to nutrient acquisition and plant development. Complementary evidence was reported by Shehata et al. [42], who showed that an endophytic bacterium associated with maize colonized root hair cells while facilitating the acquisition of phosphorus from rock phosphate. Together, these studies demonstrate that endophytic bacteria capable of phosphate mobilization can establish intimate associations with internal plant tissues and contribute to phosphorus acquisition under nutrient-limited conditions.

The ecological significance of endophytic PSB extends beyond phosphorus mobilization by influencing interactions within the plant-associated microbiome. Li et al. [39] reported that inoculation with PSB increased phosphorus availability, altered the composition of microbial communities in both the rhizosphere and endosphere, and enhanced biological nitrogen fixation in wheat. Zhu et al. [43] showed that AM fungal inoculation enhanced the recruitment of siderophore-producing PSB and promoted the solubilization of sparingly soluble inorganic P in subtropical orchard soils. AM-associated *Bradyrhizobium elkanii* strains exhibited high siderophore production and strong potential to mobilize Fe- and Ca-bound P, while AM inoculation increased P solubilization and plant biomass. The study highlights the synergistic contribution of AM fungi and PSB to improving plant P nutrition. Lu et al. [44] reported that *Burkholderia* sp. YM3 effectively solubilized calcium–magnesium–phosphate fertilizer and released soluble P, Ca^2+^, and Mg^2+^. The combined YM3 + CMP application improved P accumulation and wheat growth and alleviated Cd-induced toxicity by regulating Cd and P transporter genes. These findings suggest that phosphorus mobilization by PSB may influence microbial processes beyond phosphorus cycling through changes in the structure of plant-associated microbial communities. Although the rhizosphere is often considered a relatively homogeneous habitat, accumulating evidence indicates that substantial spatial heterogeneity exists at the microscale.

## 3. Diversity of Phosphate-Solubilizing Bacteria

PSB comprise a phylogenetically diverse group of microorganisms distributed across multiple bacterial phyla, reflecting the convergent evolution of phosphorus-solubilization strategies rather than a single evolutionary lineage [45]. Although the ability to mobilize insoluble phosphorus has been documented in numerous bacterial taxa, current evidence indicates that this functional trait is predominantly associated with members of the *phyla Bacillota* (formerly Firmicutes), *Pseudomonadota* (formerly Proteobacteria), and *Actinomycetota* (formerly Actinobacteria). These bacterial groups have been isolated from a wide range of terrestrial environments, demonstrating that phosphate solubilization is a widespread microbial function with considerable ecological and taxonomic diversity [14,24].

The taxonomic spectrum of PSB has expanded considerably during the past decade owing to advances in microbial isolation, molecular identification, and genome-based characterization. Besides the well-established plant growth-promoting genera *Bacillus*, *Pseudomonas*, *Rhizobium*, *Enterobacter*, *Burkholderia*, *Paenibacillus*, *Serratia*, and *Streptomyces*, recent investigations have identified additional taxa adapted to specialized ecological niches, including the actinobacterial genus *Paenarthrobacter* [13,46].

Collectively, these findings indicate that phosphate solubilization is not restricted to a limited number of bacterial groups but represents a functional trait that has evolved across phylogenetically distant microorganisms occupying diverse ecological habitats [47]. The composition of PSB communities varies substantially among ecosystems in response to soil physicochemical properties, vegetation type, climatic conditions, and phosphorus availability. Members of *Bacillus* and *Pseudomonas* are frequently recovered from agricultural soils and rhizosphere environments, reflecting their widespread occurrence in crop-associated microbial communities [48]. In contrast, actinobacterial genera such as *Streptomyces* are frequently associated with nutrient-limited and environmentally stressful soils, including arid and semi-arid agricultural ecosystems [49], whereas *Paenarthrobacter* species have also been reported as rhizosphere-associated plant growth-promoting bacteria possessing multiple beneficial traits, including phosphate solubilization, siderophore production, ACC deaminase activity, and indole-3-acetic acid production [50]. Nitrogen-fixing bacteria belonging to the genus *Rhizobium* contribute simultaneously to biological nitrogen fixation and phosphorus mobilization, while *Burkholderia* species have been increasingly recognized for their ability to colonize degraded ecosystems characterized by low phosphorus availability and multiple abiotic constraints [51,52,53,54].

Different PSB taxa vary in their phosphorus-solubilizing capabilities, metabolic traits, ecological adaptations, and interactions with other members of the rhizosphere microbiome. Through interactions with non-PSB microorganisms, PSB may contribute to complementary or potentially synergistic microbial functions, whereby different microorganisms produce metabolites, phytohormones, siderophores, or other compounds that collectively support nutrient acquisition, plant growth, and stress tolerance [55]. Such potential functional complementarity may contribute to the stability and resilience of rhizosphere microbial communities and provides a conceptual basis for developing multifunctional microbial inoculants tailored to different agroecosystems [22,28]. For example, Egamberdieva et al. [11] demonstrated that the co-inoculation of the phosphate-solubilizing *Pseudomonas* with the nitrogen-fixing *Mesorhizobium* enhanced liquorice growth, nodulation, and nutrient acquisition under salt stress, illustrating how microorganisms with distinct functional traits can interact to provide complementary benefits to plants. Similarly, Wani et al. [16] demonstrated under field conditions that co-inoculation of nitrogen-fixing *Mesorhizobium* with P-solubilizing *Pseudomonas* and *Bacillus* strains enhanced nutrient uptake and grain yield in chickpea. In another study, co-inoculation of *Pseudomonas fluorescens* FAP2 and *Bacillus licheniformis* B642, both possessing multiple PGPR traits, including phosphate solubilization, IAA production, and siderophore production, resulted in enhanced wheat growth and photosynthetic performance, demonstrating the potential benefits of compatible bacterial interactions within the rhizosphere and highlighting the value of combining microorganisms with complementary plant-growth-promoting functions [56].

Among the selected studies, Zhao et al. [57] characterized *Bacillus altitudinis* GQYP101, a PSB isolated from the rhizosphere of *Lycium barbarum* L. The isolate exhibited P-solubilizing activity, enhanced phosphorus accumulation in corn seedlings, increased soil-available phosphorus, and altered the rhizosphere microbial community following inoculation. Whole-genome sequencing further identified genes associated with phosphate solubilization, alkaline phosphatase activity, chemotaxis, and motility, demonstrating the value of genome-based approaches for understanding functional diversity within *Bacillus* strains.

Additional evidence indicates that P-solubilizing *Bacillus* species are not restricted to a single agricultural ecosystem. Zhang et al. [58] isolated *Bacillus* strains from mushroom residues and demonstrated their ability to promote tomato growth, whereas Gupta et al. [59] recovered *Bacillus* isolates from the rice rhizosphere during the screening of PSB. These studies illustrate that members of the genus occupy distinct ecological niches while maintaining the capacity to mobilize insoluble phosphorus.

Overall, the studies included in this review consistently identify *Bacillus* as an important PSB genus occurring in diverse agricultural environments. However, the available evidence also indicates substantial strain-level variability in P-solubilizing performance and associated plant growth-promoting characteristics, emphasizing the need for detailed taxonomic and functional characterization before selecting isolates for biofertilizer development.

Among the studies included in this review, Brito et al. [60] investigated *Paenibacillus sonchi* SBR5 using physiological experiments combined with transcriptomic analysis. The authors demonstrated that bacterial responses differed under soluble and insoluble phosphate conditions, with differential expression of genes involved in phosphorus acquisition and carbon metabolism. Their results indicate that phosphate solubilization in *P. sonchi* is a coordinated physiological response regulated by multiple metabolic pathways rather than a single mechanism.

*Pseudomonas* spp. were recovered from different ecological niches, including plant endospheres and rhizosphere soils, demonstrating that P-solubilizing members of this genus occupy diverse habitats and establish close associations with plants [23]. Among the selected studies, Oteino et al. [41] provided the most comprehensive evaluation of P-solubilizing *Pseudomonas*. The authors isolated endophytic *Pseudomonas* strains from plants and demonstrated that selected isolates were capable of solubilizing inorganic phosphate while simultaneously promoting plant growth.

Evidence from karst ecosystems further demonstrates the taxonomic occurrence of *Pseudomonas* within PSB communities. Xie et al. [52] isolated PSB belonging to the genera *Acinetobacter*, *Paraburkholderia*, and *Pseudomonas* from fruit-tree rhizosphere soils in a karst rocky desertification region. Although the detailed physiological and molecular analyses focused on *Acinetobacter* sp. Ac-14, the recovery of *Pseudomonas* isolates from these phosphorus-deficient soils indicates that members of this genus contribute to the taxonomic diversity of PSB communities under environmentally challenging conditions.

Members of the genera *Burkholderia* and *Paraburkholderia* represent important PSB adapted to diverse soil environments. Although these genera have been less extensively investigated than *Bacillus* and *Pseudomonas*, available studies demonstrate their potential for phosphorus mobilization under different environmental conditions. Chen et al. [61] isolated and molecularly identified a *Burkholderia* strain and subsequently optimized the culture conditions influencing its P-solubilizing activity. Their findings highlighted that P-solubilizing efficiency is strongly affected by environmental and nutritional conditions, emphasizing the importance of strain optimization for practical agricultural applications. Joshi et al. [62] investigated phosphate solubilization by *Rhizobium* sp. RM and RS and demonstrated that the process was influenced by the available carbon source. The authors showed that glucose and arabinose supported phosphate solubilization, whereas succinate exerted catabolite repression, indicating that P-solubilizing efficiency is closely linked to bacterial carbon metabolism. Their findings provide insight into the physiological regulation of P-solubilization in *Rhizobium*. More recently, Zhu et al. [51] isolated *Rhizobium tropici* LNP6 and demonstrated its efficient P-solubilizing ability together with plant growth-promoting effects on *Ormosia hosiei* seedlings. The study confirmed that inoculation with the strain enhanced seedling growth while improving phosphorus availability, supporting the potential application of PSB *Rhizobium* in forestry and ecological restoration.

Species of the genus *Enterobacter* possess P-solubilizing ability and can improve plant phosphorus nutrition under different soil conditions. Chen et al. [63] characterized PSB isolated from maize cultivated in calcareous soils of Sinaloa, Mexico, and identified *Enterobacter* among the bacterial taxa exhibiting plant growth-promoting potential. The isolates were selected based on P-solubilizing activity and evaluated for their capacity to improve phosphorus nutrition in maize, highlighting the occurrence of *Enterobacter* in calcareous agricultural soils. Mohamed et al. [64] isolated PSB from the wheat rhizosphere in Egyptian calcareous soils and identified *Enterobacter aerogenes* and *Enterobacter* sp. among the most efficient isolates. Both strains exhibited high tricalcium P-solubilizing activity and IAA production, while greenhouse experiments demonstrated significant improvements in wheat growth, phosphorus uptake, and soil-available phosphorus following bacterial inoculation. Safirzadeh et al. [65] further demonstrated the agricultural potential of P-solubilizing *Enterobacter* by evaluating *Enterobacter cloacae* as a bioinoculant for sugarcane. The study reported improved phosphorus uptake and plant performance following bacterial inoculation, supporting the applicability of *Enterobacter* as a microbial inoculant across different cropping systems.

Yaling et al. [66] reported that *Pediococcus pentosaceus* PSM16 markedly enhanced soil P availability, increasing effective P from 0.270 to 2.109 mg kg^−1^, while increasing the proportion of available P to total P by nearly 40%. PSM16 also enhanced P availability when applied with P fertilizer, whereas fertilizer alone resulted in only 0.105 mg kg^−1^ available P. Furthermore, functional analysis showed that PSM16 phosphatase-related genes increased acid phosphatase activity to up to 11,267 nmol d^−1^ g^−1^ and increased soil-available P to 3.74 mg kg^−1^, demonstrating the strong P-solubilizing potential of this strain. In another study, Kouas et al. [67] isolated 47 bacterial strains from arid soils in southern Tunisia and identified several salt-tolerant PSB with strong P-solubilizing and IAA-producing abilities. The most effective strains released up to 126.8 μg P mL^−1^ after 7 days and produced up to 101.86 μg IAA mL^−1^ after 3 days. Inoculation of barley under P deficiency significantly enhanced biomass, shoot and root growth, chlorophyll content, photosynthetic activity, P uptake and translocation, as well as P absorption and utilization efficiencies, highlighting the potential of native PSB from saline and arid environments to improve plant P nutrition under P-limited conditions.

Species of the genus *Serratia* have been reported as PSB in diverse terrestrial environments, including agricultural soils and P-rich substrates, where they have been evaluated for their potential to enhance phosphorus availability and plant growth. Guo et al. [68] isolated a P-tolerant *Serratia* sp. (LX2) capable of solubilizing rock phosphate under different phosphorus concentrations. The study demonstrated efficient P-solubilization accompanied by a decline in medium pH, indicating the strain’s potential for mobilizing poorly soluble phosphate sources under phosphorus-limited conditions. Similarly, Zheng et al. [69] investigated *Serratia marcescens* N1.14 X-45 as a rock PSB associated with legume growth. The strain significantly enhanced plant biomass and phosphorus uptake in pot experiments while improving soil-available phosphorus. The study also reported changes in rhizosphere microbial community composition following bacterial inoculation, suggesting that phosphate solubilization was accompanied by broader effects on the rhizosphere environment. Gong et al. [70] characterized *Serratia marcescens* Pt-3 as a bacterium possessing both P-solubilizing activity and antifungal volatile production. In addition to solubilizing inorganic phosphate, the strain produced volatile organic compounds that inhibited the growth of several phytopathogenic fungi, highlighting its potential as a multifunctional microbial inoculant for sustainable crop production.

Species of the genus *Streptomyces* have attracted considerable attention as P-solubilizing actinobacteria because of their capacity to mobilize insoluble phosphorus and improve plant phosphorus acquisition under different soil conditions. Aallam et al. [54] evaluated phosphate- and potassium-solubilizing *Streptomyces* isolates associated with sugar beet. The authors demonstrated that selected strains were able to solubilize both tricalcium phosphate and rock phosphate and, under greenhouse conditions, promoted sugar beet growth while reducing the severity of *Fusarium* root rot. The study also showed considerable variation in P-solubilizing efficiency among the tested Streptomyces isolates, emphasizing the strain-dependent nature of these traits [56].

Cui et al. [71] investigated the endophytic strain *Streptomyces* sp. CoT10 isolated from *Camellia oleifera*. Unlike most PSB that are evaluated primarily for calcium phosphate dissolution, CoT10 effectively mobilized Fe-bound phosphate, with the study demonstrating that this activity was directly mediated by siderophore production. Inoculation with CoT10 increased soil-available phosphorus, enhanced phosphorus and iron acquisition by the host plant, and promoted plant growth. In addition, the authors reported shifts in rhizosphere bacterial diversity and predicted phosphorus- and iron-related microbial functions following inoculation. Biswas et al. [72] demonstrated that PSB-activated low-grade rock phosphate (RP) can serve as a sustainable alternative to conventional P fertilizers. In soil incubation, PSB-activated RP progressively increased plant-available P to levels comparable with diammonium phosphate (DAP). In a wheat pot experiment, PSB-activated RP replaced 50% of conventional P fertilizer without reducing yield or P uptake. The combined application of DAP, RP, and PSB also improved soil functional diversity and enzyme activities, indicating that PSB-assisted RP can reduce conventional P fertilizer use while maintaining crop productivity and improving soil biological properties.

Collectively, these studies demonstrate that PSB strains can mobilize chemically distinct insoluble phosphorus pools, including calcium- and iron-bound phosphates, thereby improving plant phosphorus nutrition under different environmental conditions.

## 4. Mechanisms of Phosphate Solubilization and Mineralization by PSB

**Figure 2:**
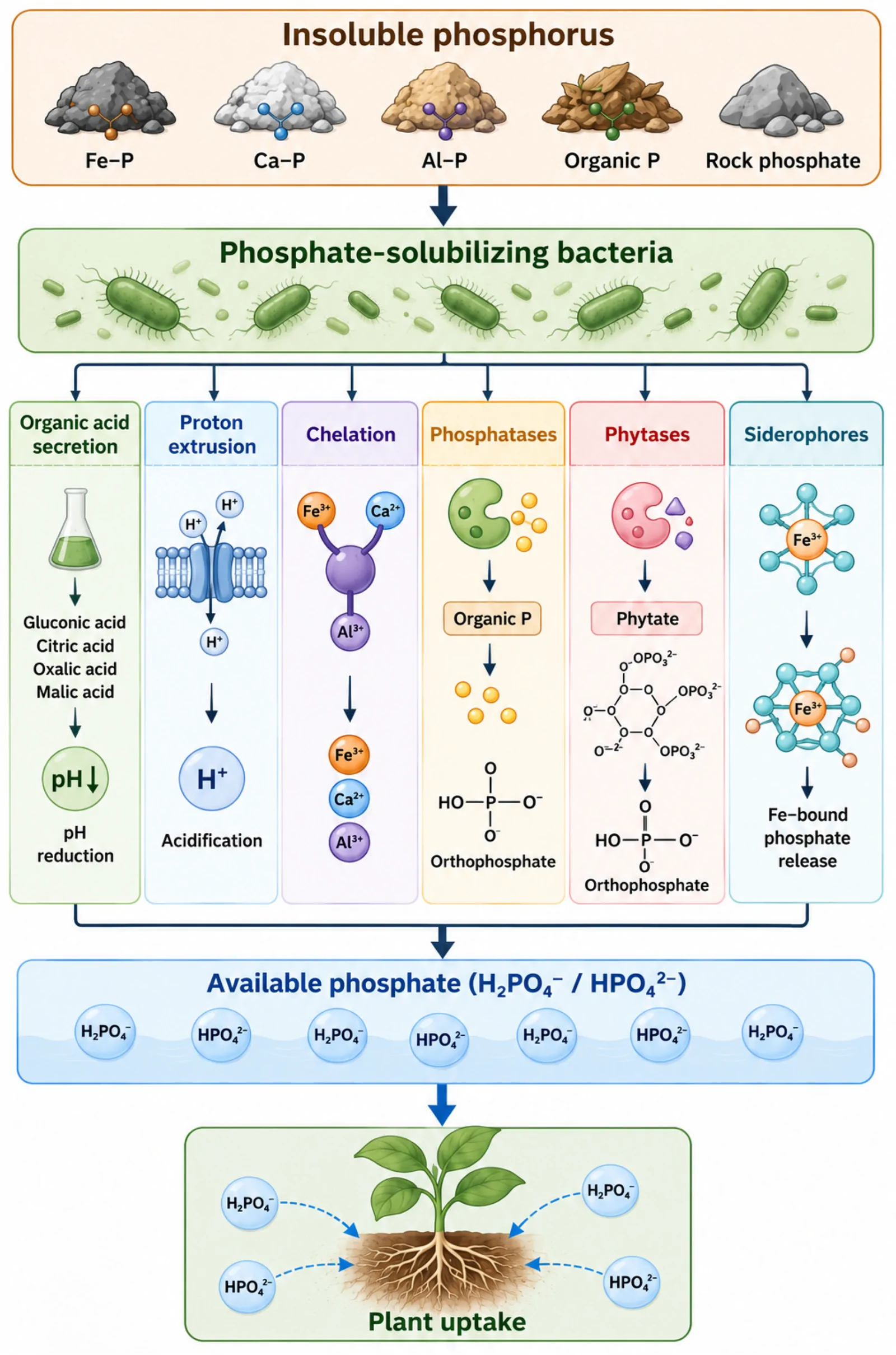
Major mechanisms of phosphorus solubilization and mineralization by PSB.

PSB mobilize poorly available soil phosphorus through multiple complementary mechanisms that collectively increase the availability of orthophosphate for plant uptake. These mechanisms target both inorganic phosphorus pools, including calcium-, iron-, and aluminum-associated phosphates, and organic phosphorus compounds that require enzymatic mineralization before becoming plant-available [7,19]. Phosphorus mobilization by PSB results from the coordinated operation of multiple mechanisms rather than a single universal process, including organic acid production, proton extrusion, metal chelation, phosphatase- and phytase-mediated hydrolysis, and siderophore production, extracellular polymeric substances, together with effective rhizosphere colonization that supports sustained phosphorus mobilization. The relative contribution of each mechanism depends on bacterial genotype, phosphorus source, soil physicochemical properties, and rhizosphere conditions [66,67,68]. Figure 2 provides a conceptual overview of the principal mechanisms involved in phosphorus solubilization and mineralization by PSB, which are discussed in detail in the following sections.

### 4.1. Organic Acid-Mediated Solubilization of Inorganic Phosphate

The dominant forms of inorganic phosphorus vary with soil pH and mineral composition. Calcium phosphates predominate in neutral to alkaline soils, whereas in acidic soils phosphorus is mainly associated with iron and aluminum minerals through precipitation or strong surface adsorption. Phosphorus speciation analyses based on X-ray absorption spectroscopy and sequential fractionation have consistently shown that a substantial proportion of soil phosphorus occurs in mineral-associated forms with limited solubility under natural soil conditions [66,73]. Consistent with these findings, rhizosphere phosphorus fractionation analyses have identified poorly available inorganic phosphorus as the dominant fraction in the studied acidic soils, underscoring the importance of biological processes that mobilize mineral-bound phosphorus into plant-available orthophosphate [74,75,76]. These sparingly soluble mineral phosphorus pools therefore constitute the principal targets of PSB during inorganic phosphate solubilization.

Among the microbial processes involved in inorganic phosphate solubilization, the extracellular secretion of low-molecular-weight organic acids is widely recognized as a principal mechanism driving phosphorus mobilization [19]. Multi-omics analysis by Ding et al. [77] showed that growth under insoluble phosphate conditions was accompanied by increased production of several organic acids, a decline in medium pH, higher concentrations of soluble phosphorus, and differential expression of genes involved in organic acid metabolism, collectively indicating a close association between organic acid production and phosphate solubilization. Comparative analyses revealed substantial variation in the composition and quantity of organic acids produced by phylogenetically distinct P-solubilizing bacteria, indicating that diverse metabolic strategies can achieve efficient phosphate solubilization. Community-level evidence further links enhanced organic acid production during PSB inoculation with increased phosphorus release during composting, highlighting the contribution of acid-mediated phosphorus mobilization under complex environmental conditions [78]. Following their secretion, organic acids promote inorganic phosphate solubilization primarily through proton-mediated acidification of the surrounding microenvironment. Dissociation of carboxyl groups releases H^+^ ions, thereby lowering the local pH and increasing the dissolution of sparingly soluble phosphate minerals, particularly calcium phosphates. Using *Pseudomonas aeruginosa* P4, Buch et al. [79] demonstrated that phosphorus deficiency redirected glucose metabolism toward gluconate production, linking enhanced organic acid biosynthesis with P-solubilizing activity. Similarly, Vyas and Gulati [80] reported that increased organic acids production by P-solubilizing fluorescent *Pseudomonas* strains was accompanied by greater acidification of the growth medium and increased phosphorus release.

Although proton-mediated acidification plays a central role in phosphate dissolution, it does not fully explain differences in the solubilization of various phosphate minerals. Organic acids also contribute through metal chelation and ligand exchange, whereby carboxyl groups form stable complexes with cations such as Ca^2+^, Fe^3+^, and Al^3+^, thereby destabilizing mineral structures and facilitating phosphate release. Under buffered conditions designed to minimize pH effects, Kaur et al. [81] showed that PSB produced distinct organic acid profiles in response to different insoluble phosphate sources, resulting in variable solubilization efficiencies. Likewise, Mendes et al. [82] demonstrated that oxalic acid dissolved rock phosphate more effectively than sulfuric acid despite comparable acidity, highlighting the importance of ligand properties beyond proton release [83]. Consistent with these observations, Zuluaga et al. [84] showed that organic acids produced by *Enterobacter* sp. strain 15S enhanced tricalcium phosphate solubilization in the cucumber rhizosphere. Together, these studies indicate that inorganic phosphate solubilization is mediated by both proton-induced acidification and specific interactions between organic acid ligands and mineral-associated metal cations.

Although organic-acid production is frequently demonstrated in culture-based assays, evidence from soil–plant systems indicates that organic acids can also contribute to P mobilization in the rhizosphere. Panhwar et al. [85] evaluated a *Bacillus* PSB strain with oxalic or malic acid and phosphate rock under aerobic rice cultivation. Application of 20 mM oxalic acid with PSB16 increased soluble soil P to 28.39 mg kg^−1^, plant P uptake to 0.78 P pot^−1^, and plant biomass to 33.26 mg. The bacterial population was also higher in the rhizosphere (8.78 log_10_ CFU g^−1^) than in the non-rhizosphere and endosphere. Notably, the combined treatment did not significantly alter soil pH, indicating that P mobilization under soil conditions cannot be attributed solely to bulk-soil acidification and may instead involve localized organic-acid activity and microbial–root interactions. Kaur and Reddy [86] isolated PSB from an organic farming field and selected *Pantoea cypripedii* and *Pseudomonas plecoglossicida* based on their phosphate-solubilizing activity, organic-acid production, and phosphatase and phytase activities. Field inoculation of maize with these strains increased soil-available P and total plant P uptake, accompanied by improvements in maize yield, soil organic carbon, and soil enzyme activities.

Organic acid production is a regulated metabolic response rather than an inherent trait of PSB. In many Gram-negative bacteria, phosphate solubilization is closely associated with the pyrroloquinoline quinone (PQQ)-dependent glucose dehydrogenase (Gcd) pathway, in which PQQ-dependent glucose dehydrogenase catalyzes the oxidation of glucose to gluconic acid. Buch et al. [80] showed that phosphorus deficiency redirected glucose metabolism toward gluconate production in *Pseudomonas aeruginosa* P4, providing a metabolic basis for enhanced P-solubilization. Similarly, An and Moe [87] demonstrated that PQQ-dependent glucose dehydrogenase activity in *Pseudomonas putida* KT2440 is regulated by environmental and physiological conditions. Moreover, Patel et al. [88] reported that exogenous weak organic acids repressed the P-solubilizing phenotype of fluorescent *Pseudomonas*. Together, these studies indicate that efficient phosphorus mobilization depends not only on the capacity to synthesize organic acids but also on the regulatory mechanisms controlling their production.

### 4.2. Enzymatic Mineralization of Organic Phosphorus

Organic phosphorus constitutes a substantial fraction of total soil phosphorus and occurs in diverse compounds, including inositol phosphates, phospholipids, nucleic acids, and phosphoproteins, many of which are not directly available for plant uptake [89]. Before plants can utilize these phosphorus forms, they must be hydrolyzed into inorganic orthophosphate through the action of extracellular enzymes produced primarily by soil microorganisms, including phosphatases and phytases [7]. Consequently, microbial enzymatic mineralization represents a key biological process regulating phosphorus recycling and the pool of potentially plant-available phosphorus in terrestrial ecosystems. Recent advances in comparative genomics, molecular ecology, and biochemical characterization have substantially expanded our understanding of the bacterial enzymes responsible for organic phosphorus mineralization and their ecological distribution.

The enzymatic mineralization of organic phosphorus is mediated by several phosphatase systems that differ in their evolutionary origin, taxonomic distribution, and regulatory characteristics. Comparative genomic analysis by Sebastián and Ammerman [90] showed that the alkaline phosphatase PhoX is more widely distributed than the classical PhoA among marine bacteria, providing evidence of the genetic potential for phosphatase-mediated phosphorus acquisition rather than direct evidence of phosphorus transformation. In soils, Ragot et al. [91] revealed extensive phylogenetic diversity of phoD-harboring bacteria, while metagenomic analyses by Garaycochea et al. [92] identified phoD as one of the most abundant phosphorus-cycling genes, indicating widespread genetic potential for organic phosphorus mineralization but not necessarily expression of the encoded enzyme or realized mineralization under field conditions. More recently, Lidbury et al. [93] identified and biochemically characterized phosphate-insensitive phosphatase A (PafA) using comparative genomics together with gene knockout, complementation, and enzymatic assays. Unlike the classical phosphate-starvation phosphatases, PafA retained phosphatase activity independently of phosphate availability, providing direct biochemical evidence of enzyme function and a mechanistic basis for bacterial organophosphorus remineralization. Collectively, these studies provide different levels of evidence, ranging from genetic potential to experimentally verified enzyme activity, and these should not be interpreted as equivalent to measured phosphorus transformation in soil or subsequent plant phosphorus uptake [94].

The contribution of phosphatase-producing microorganisms to organic phosphorus mineralization depends on environmental conditions that shape microbial community composition and phosphatase functional potential. At the rhizosphere scale, Guo et al. [95] and Fraser et al. [96] demonstrated that plant roots strongly influenced phosphatase gene abundance, phosphatase activity, and phosphorus-mineralizing bacterial communities. Likewise, ecosystem development altered phosphatase-related microbial communities, as shown during restoration of mining-degraded soils, and biological soil crust succession [27]. However, because most studies rely on gene abundance, community composition, or bulk enzyme activity, they reveal ecological patterns rather than direct causal relationships between specific microorganisms and organic phosphorus mineralization [97,98].

In addition to non-specific phosphatases, phytases play an important role in bacterial organic phosphorus mineralization by hydrolyzing phytate, one of the major organic phosphorus compounds in many agricultural soils. Lim et al. [99] showed that phytate-mineralizing bacteria are distributed across diverse bacterial taxa, although the capacity for phytate utilization varied considerably among isolates. More recently, Zhou et al. [100] reported that combined application of phytase and PSB increased soil phytase activity, phosphorus availability, and maize phosphorus uptake in low-phosphorus red soil. These findings support the contribution of phytase-mediated mineralization to phosphorus acquisition under phosphorus-deficient conditions, although the relative importance of microbial phytases under field conditions remains to be clarified.

Long-term organic fertilization altered the composition of phoD- and pqqC-harboring bacterial communities while increasing phosphorus availability, indicating that sustained management can reshape microbial communities involved in both organic phosphorus mineralization and inorganic phosphate solubilization [73]. Likewise, He et al. [101] identified core phoD-harboring taxa positively associated with alkaline phosphatase activity and wheat phosphorus uptake, although these relationships were based on ecological associations rather than direct functional evidence. Under controlled soil conditions, Wu et al. [102] demonstrated that phosphorus-activating amendments increased phosphatase activities together with available phosphorus, providing direct biochemical evidence of enhanced phosphorus transformation.

Despite substantial advances in characterizing phosphatase-producing microorganisms, important gaps remain in understanding how microbial functional potential is translated into organic phosphorus mineralization under natural conditions. Current studies have greatly expanded knowledge of phosphatase diversity, environmental regulation, and the ecological distribution of phosphorus-mineralizing microorganisms [103]. However, relatively few investigations have simultaneously examined microbial community composition, phosphatase genes, enzyme activity, phosphorus transformation, and plant phosphorus acquisition within the same experimental framework. Consequently, direct mechanistic links between microbial functional potential and ecosystem-scale phosphorus cycling remain incompletely resolved. As highlighted in the synthesis by Pang et al. [104], efficient soil phosphorus mobilization results from the coordinated activities of PSB, phosphorus-mineralizing microorganisms, plant roots, and soil physicochemical processes rather than from any single microbial mechanism [105]. The principal biochemical mechanisms by which PSB mobilize inorganic and organic phosphorus into plant-available orthophosphate are summarized in Figure 2.

### 4.3. Siderophore-Mediated Mobilization

Siderophores are secondary metabolites produced by bacteria and fungi under iron-limited conditions to acquire ferric iron (Fe^3+^). Owing to their high affinity for Fe^3+^, they can indirectly mobilize phosphorus by chelating iron associated with Fe-bound phosphorus, thereby increasing phosphorus availability [106,107]. Unlike organic acid-mediated dissolution or phosphatase-mediated mineralization, siderophore-mediated phosphorus mobilization relies on Fe^3+^ sequestration, which weakens Fe–P associations and promotes phosphorus release [108]. Ghosh et al. [109] demonstrated that siderophore-producing rhizobacteria enhanced FePO_4_ solubilization and increased plant phosphorus uptake, indicating that siderophore production promotes phosphorus acquisition from iron phosphate. Together, these studies demonstrate that siderophore production facilitates the mobilization of phosphorus associated with iron-bearing mineral phases. Wang et al. [110] demonstrated a mechanism underlying siderophore-mediated phosphorus mobilization by showing that Fe complexation inhibited phytase-mediated degradation of organic phosphorus. The siderophore ferrichrome removed Fe^3+^ from Fe–phytate complexes, restoring phytase accessibility and increasing phosphorus release. These findings indicate that siderophores indirectly enhance organic phosphorus mobilization by disrupting Fe–phytate complexes rather than directly hydrolyzing phosphorus compounds.

### 4.4. Extracellular Polymeric Substance-Mediated Mobilization

Extracellular polymeric substances (EPS) are extracellular matrices composed primarily of polysaccharides, proteins, nucleic acids, and lipids that form the structural framework of microbial biofilms. Their abundant carboxyl, hydroxyl, and amino groups enable interactions with mineral surfaces, metal ions, and dissolved compounds, thereby influencing nutrient dynamics and mineral transformations [111,112]. In soils, EPS create a microbe–mineral interface that regulates mineral–organic interactions and nutrient cycling, providing the physicochemical basis for EPS-mediated phosphorus mobilization [113].

Luo et al. [114] demonstrated that EPS regulate mineral surface processes governing phosphorus release. Using calcium phosphate as a model mineral, they showed that EPS promoted phosphorus adsorption, crystal encapsulation, and dissolution–recrystallization, altering crystal morphology without directly dissolving phosphate minerals. Similarly, Xie et al. [115] reported that purified exopolysaccharides from *Rhizobium tropici* enhanced the release of legacy phosphorus associated with iron oxides, aluminum oxides, and clay minerals through direct interactions with mineral surfaces. Because purified polysaccharides alone increased mineral-associated phosphorus availability, these findings indicate that EPS facilitate phosphorus mobilization by modifying the mineral–microbe interface rather than through direct mineral dissolution.

In addition to modifying mineral surface reactions, EPS regulate phosphorus availability through reversible adsorption–desorption processes. Ni et al. [116] showed that reactive functional groups within the EPS matrix reversibly bind phosphorus, buffering its availability at the mineral–microbe interface. Beyond phosphorus mobilization, EPS also contribute to mineral transformation and biomineralization. Sánchez-Román et al. [117] demonstrated that microbial cells and their associated EPS act as nucleation templates for Fe-rich phosphate and carbonate minerals, while Jiang et al. [118] showed that EPS produced by *Ochrobactrum* sp. promoted pyromorphite nucleation following phosphate release. Together, these studies indicate that EPS regulate both the short-term availability and long-term stabilization of phosphorus through adsorption–desorption processes and mineral transformation.

### 4.5. Rhizosphere Colonization and Biofilm Formation

Rhizosphere colonization is an ecological mechanism that enables PSB to establish persistent associations with plant roots, facilitating sustained phosphorus solubilization and mineralization in the rhizosphere. The rhizosphere is the soil zone where plants interact with microorganisms through the release of root exudates, which serve as nutrient sources and signaling molecules that shape microbial community assembly [119]. These exudates, consisting of sugars, amino acids, organic acids, phenolics, flavonoids, and other metabolites, not only provide nutrients but also function as chemical signals that influence microbial community assembly. Under phosphorus (P) deficiency, plants modify both the composition and quantity of root exudates, selectively enriching microorganisms capable of improving P acquisition, including PSB [120]. This adaptive response highlights the role of root exudates as ecological filters that selectively enrich beneficial microorganisms, including PSB, rather than simply stimulating overall microbial growth.

Chemotaxis is the first step in rhizosphere colonization, with root-derived metabolites establishing chemical gradients that guide motile bacteria toward actively growing roots, particularly the root apex where exudation is greatest [121,122]. Recruitment is highly selective because individual exudate components differ in their attractiveness to bacterial taxa; for example, amino acids such as arginine act as strong chemoattractants for several beneficial rhizobacteria, whereas other metabolites exhibit species-specific effects [123]. Consistent with this, O’Neal and Alexandre [124] showed that efficient chemotaxis is essential for successful root colonization, demonstrating that rhizosphere recruitment is directed by plant-derived chemical cues rather than random microbial encounters.

Beyond attracting microorganisms, root exudates also regulate bacterial function. Pantigoso et al. [37] showed that metabolites enriched in phosphorus-deficient root exudates enhanced P-solubilizing activity without substantially increasing bacterial growth, suggesting that plants selectively activate microbial functions required for phosphorus acquisition. Likewise, P-deficient exudates preferentially enriched bacterial groups associated with phosphorus mobilization, indicating that plants recruit microbial functions as well as microbial taxa. Together, these findings show that root exudates shape both the composition and functional capacity of the rhizosphere microbiome.

Successful recruitment alone, however, does not ensure effective plant PSB interactions. Recruited bacteria must compete with indigenous microorganisms and efficiently utilize root-derived substrates to establish persistent populations. Fourneau et al. [125] demonstrated that attraction to root exudates and their utilization are complementary but independent traits, whereas De Zutter et al. [126] identified rhizosphere competence as a key determinant of successful establishment. Thus, root exudate-mediated recruitment is a multistage process linking chemotaxis, functional activation, and stable rhizosphere colonization.

Following chemotactic recruitment, successful plant PSB interactions depend on stable colonization of the root surface. Fluorescence-based studies showed that bacterial cells preferentially colonize root tips, elongation zones, and emerging root hairs, where bacterial abundance is greater than on older root tissues, reflecting the spatial heterogeneity of the rhizosphere [40,127,128]. Root colonization is a dynamic process that accompanies continued root growth, requiring bacterial populations to maintain associations with newly developed tissues over time [129]. Although colonization efficiency varies among bacterial strains and host species, stable rhizoplane colonization is an essential prerequisite for persistent plant PSB interactions and subsequent endophytic establishment [20,130].

While many PSB remain associated with the rhizoplane, certain strains can colonize internal root tissues and establish endophytic populations. Fluorescence microscopy combined with bacterial re-isolation showed a temporal progression from root surface colonization to internal cortical tissues by *Bacillus cereus* YL6 in Chinese cabbage, supporting the view that endophytic establishment follows successful root surface colonization [40]. Comparable endophytic associations have also been reported in maize and Populus, indicating that internal colonization can occur across multiple host species when compatible plant–microbe interactions are established [131,132]. Current evidence further suggests that endophytic competence is strain dependent, with successful internal establishment varying among bacterial isolates and host plants rather than representing a universal characteristic of all PSB [44,133].

Experimental evidence indicates that stable colonization is associated with enhanced phosphorus uptake, modifications in root system architecture, and improved plant growth across diverse cropping systems [134,135,136,137,138]. Beyond improvements in phosphorus nutrition, transcriptomic analyses have shown that persistent colonization can alter the expression of genes involved in nutrient transport, root development, and stress responses, demonstrating that prolonged bacterial residence within root tissues is accompanied by coordinated changes in plant physiological processes. Similarly, under environmentally challenging conditions, stable PSB colonization has been associated with modifications in rhizosphere microbial communities and improved plant performance, suggesting that persistent plant–microbe associations contribute to enhanced ecological resilience [44]. Table 1 summarizes the major phosphorus-solubilization mechanisms reported for representative PSB species.

### 4.6. Phosphate-Solubilizing Bacteria as Drivers of Plant Nutrition, Growth, and Stress Resilience

One of the most consistently reported physiological consequences of PSB interactions is the improvement in plant phosphorus nutrition, which provides the foundation for enhanced growth and productivity [143]. Across diverse cropping systems, including maize, rice, and tomato, PSB inoculation has been consistently associated with increased phosphorus availability in the rhizosphere, resulting in greater phosphorus accumulation in plant tissues and improved vegetative growth under both controlled and field conditions [65,125,144,145].

These improvements in plant phosphorus status are frequently accompanied by increased biomass production and enhanced crop performance, indicating that mobilized phosphorus is effectively utilized to support plant development rather than remaining unavailable in the soil [146]. Importantly, evidence from field experiments demonstrates that these physiological benefits extend beyond early vegetative growth to agronomically relevant outcomes. In maize, PSB inoculation improved phosphorus acquisition, plant performance, and grain yield under reduced phosphorus fertilization, whereas in tomato, inoculation enhanced fruit yield and quality while simultaneously increasing soil-available phosphorus and alkaline phosphatase activity [147]. Although the magnitude of these responses varies among bacterial taxa, host species, soil characteristics, and cultivation systems, comparable improvements have been reported for phylogenetically distinct PSB belonging to the genera *Rhizobium*, *Pseudomonas*, *Acinetobacter*, *Burkholderia*, and *Bacillus*, suggesting that enhanced phosphorus nutrition represents a conserved functional outcome of successful plant PSB associations rather than a characteristic of individual strains [148]. Moreover, evidence that increased phosphorus mobilization can occur without increasing phosphorus losses under planted conditions suggests that efficient PSB inoculation may improve phosphorus-use efficiency while reducing environmental phosphorus losses [149]. Collectively, these studies demonstrate that enhanced phosphorus acquisition is among the most consistent physiological responses to PSB inoculation and provides the nutritional basis for subsequent improvements in plant growth, crop productivity, and agricultural performance.

Beyond their role in phosphorus mobilization, PSB can also influence plant–soil interactions under abiotic stress conditions, including heavy-metal contamination, through changes in rhizosphere chemistry, microbial communities, and metal mobility. Zhang et al. [150] found that inoculation with *Providencia rettgeri* altered rhizosphere P-cycling microbial communities and soil processes in Cd-contaminated acidic soil. Although inoculation increased Cd retention in the rhizosphere, it reduced Cd translocation and bioconcentration in *Brassica napus*. The response was mainly associated with changes in soil chemical properties and enzyme activities, highlighting the role of PSB-mediated rhizosphere processes in limiting Cd accumulation. Whereas, Luo et al. [139] demonstrated that *Enterobacter* sp. FM-1 enhanced P availability and cycling in the rhizosphere of *Solanum nigrum* under Cd contamination. PSB inoculation increased soil-available P and bioavailable Cd, which promoted Cd accumulation in the hyperaccumulator while enhancing antioxidant defense and phytochelatin production. The results highlight the coupling between microbial P activation, plant P supply, and Cd phytoremediation. Jiang et al. [140] demonstrated that PSB inoculation improved Pb phytoremediation by *Cynodon dactylon* under Pb-contaminated conditions. *Pantoea rwandensis* and *Acinetobacter calcoaceticus* enhanced soil-available P and the acid-soluble Pb fraction, while promoting plant biomass and chlorophyll content. PSB inoculation also increased Pb bioaccumulation and phytoremediation efficiency, indicating a multifunctional role of PSB in nutrient mobilization and heavy-metal remediation.

Following improvements in phosphorus availability, PSB also modify root system architecture, enabling plants to explore soil more effectively for immobile phosphorus. Experimental studies have shown that PSB inoculation promotes root developmental plasticity through increases in root length, lateral root formation, and root hair development, thereby expanding the absorptive surface available for nutrient acquisition. Because phosphorus diffuses slowly through soil, these morphological adjustments complement microbial phosphorus solubilization by increasing the likelihood of root contact with newly mobilized phosphate. For example, inoculation with PSB strains enhanced root growth and biomass in rice under phosphorus-deficient conditions, accompanying improved phosphorus uptake and overall plant performance [151,152,153]. Similarly, transcriptome-guided physiological analyses in Arabidopsis thaliana demonstrated that inoculation with a phosphate-accumulating *Pseudomonas putida* strain stimulated root branching and root hair formation under phosphorus starvation, indicating that PSB-induced changes in root morphology represent an important component of plant adaptation to phosphorus limitation [154]. While several PSB also produce auxins and other plant growth-promoting compounds associated with enhanced seedling establishment [76], not all responses involve greater root proliferation. For instance, inoculation with specific seed endophytic bacterial consortia increased soil-available phosphorus while reducing the root-to-shoot ratio under phosphorus-deficient conditions, suggesting that improved phosphorus availability may decrease the need for preferential carbon allocation to root foraging rather than simply stimulating root growth.

Consistent with this adaptive response, inoculation with efficient PSB has been reported to enhance crop growth and nutrient acquisition under both controlled and field conditions [155,156,157]. However, PSB-mediated root responses are not universally characterized by greater root proliferation. Kim et al. [148] showed that inoculation with specific seed endophytic bacterial consortia increased soil-available phosphorus while reducing the root-to-shoot ratio under phosphorus-deficient conditions, suggesting that alleviation of phosphorus limitation may reduce the need for preferential carbon allocation to root foraging rather than simply promoting continued root growth.

PSB-mediated benefits may also extend to plant responses under climate-related abiotic stresses. Under drought and salinity, PSB can contribute to stress tolerance by sustaining phosphorus and nutrient acquisition and supporting plant physiological performance under adverse conditions [158,159]. Broader evidence from plant growth-promoting bacteria indicates that the modulation of stress-related phytohormones and activation of antioxidant defenses can limit reactive oxygen species (ROS)-induced damage and help maintain photosynthetic performance under stress [160]. In PSB specifically, halotolerant strains have been shown to improve plant P status and photosynthetic activity, while drought-tolerant PSB can improve P availability and crop performance under water-deficit conditions [161,162]. PSB-associated root development, extracellular polymeric substance production, biofilm formation, and rhizosphere interactions may further support microbial persistence and nutrient acquisition under unfavorable environmental conditions [163]. However, these responses are strain-, host-, and environment-dependent, and mechanistic evidence under field conditions remains less extensive than that for PSB-mediated phosphorus mobilization and plant P acquisition [143].

**Figure 3:**
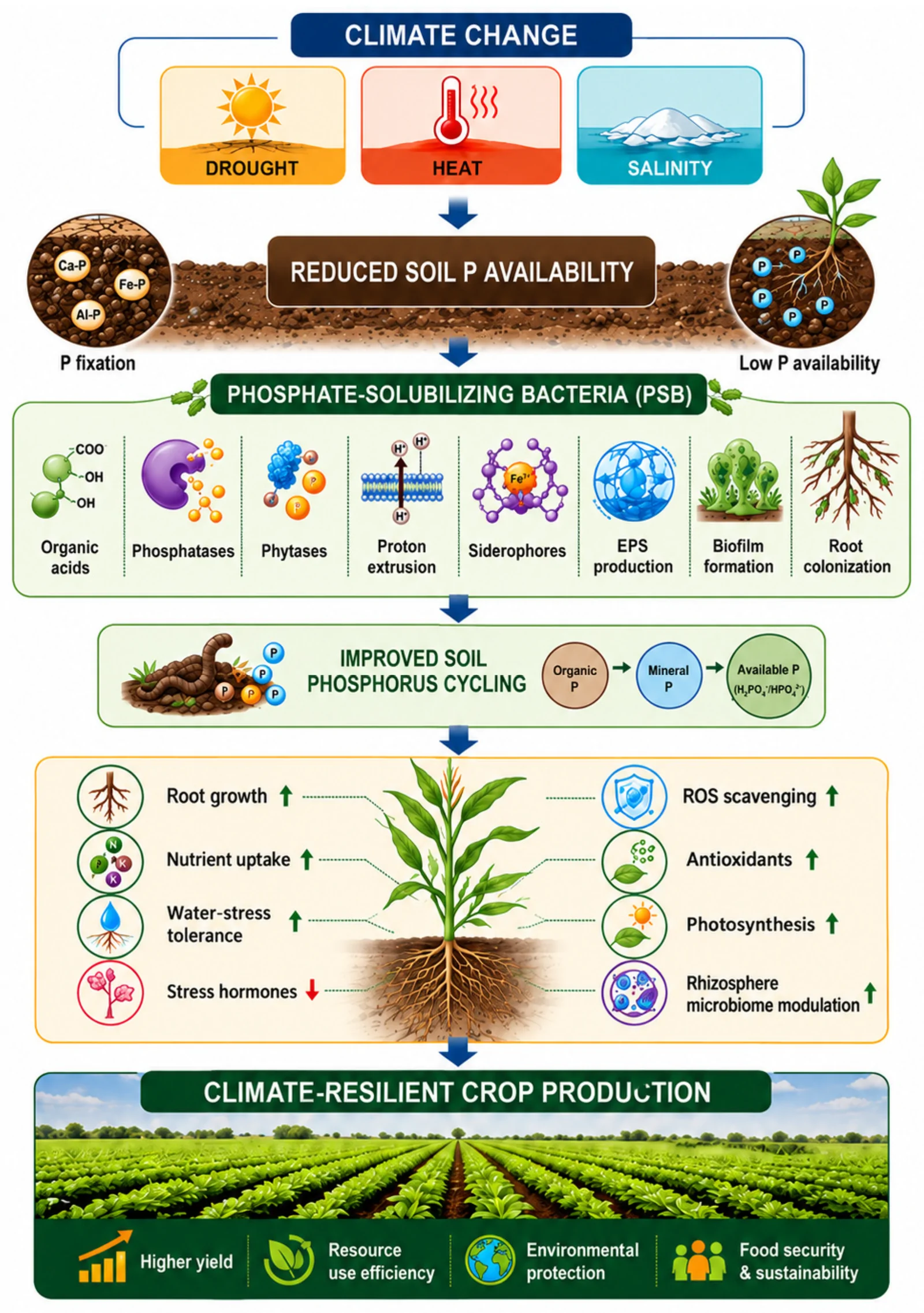
Role of PSB in enhancing plant nutrition and climate resilience.

Collectively, these findings indicate that PSB regulate root developmental plasticity in a context-dependent manner, enabling plants to adjust root architecture according to phosphorus availability and thereby optimize phosphorus acquisition under nutrient-limited conditions. The integrated role of PSB in enhancing phosphorus availability, improving plant physiological performance, mitigating abiotic stress, and promoting climate-resilient crop production is summarized in Figure 3.

## 5. Economic Aspects and Commercialization of PSB

The successful adoption of PSB in sustainable agriculture depends not only on their biological effectiveness but also on their economic feasibility and commercial viability. While considerable research has focused on the mechanisms of phosphorus mobilization and plant growth promotion, comparatively less attention has been given to the economic benefits, market development, commercialization challenges, and long-term adoption of PSB-based biofertilizers. Evaluating these aspects is essential for translating laboratory and field research into practical agricultural solutions that are economically attractive to farmers and scalable for commercial production. The following sections summarize the current economic advantages of PSB, recent advances in biofertilizer commercialization, existing constraints to market adoption, and future opportunities for integrating PSB into climate-smart and precision agricultural systems [164,165].

The economic value of PSB extends beyond their direct contribution to phosphorus mobilization by improving the overall efficiency of nutrient management in agricultural systems. Because a substantial proportion of applied phosphate fertilizers rapidly becomes immobilized through precipitation or adsorption reactions in soil, conventional phosphorus fertilization is often characterized by low recovery efficiency and high production costs [166,167]. By increasing the availability of native and fertilizer-derived phosphorus, PSB can improve phosphorus-use efficiency (PUE), enabling crops to achieve comparable or higher productivity with reduced fertilizer inputs.

Sun et al. [167] isolated PSB strains, among which included *Enterobacter* sp. AJ14 which showed the highest P-solubilizing ability, releasing P from tri-calcium phosphate, rock phosphate, ferric phosphate, and aluminum phosphate. Inoculation with its liquid formulations increased soil-available P, plant dry matter, and P uptake in green gram compared with the control and solid formulation, demonstrating a positive effect of PSB inoculation on P availability and plant productivity. Khuong et al. [142] reported that three *Enterobacter asburiae* strains showed high P-solubilizing activity, releasing up to 74.1 mg Al-P/L, 42.2 mg Ca-P/L, and 98.0 mg Fe-P/L after five days. PSRB inoculation increased soil-soluble P by 7.1 mg kg^−1^, P uptake by maize by 31%, and grain yield by 20.2%, demonstrating their potential to enhance P availability and crop productivity. Biswas et al. [72] demonstrated that PSB-activated low-grade rock phosphate could replace 50% of conventional P fertilizer without compromising wheat yield or P uptake. The DAP30 + RP60 + PSB treatment also significantly improved soil functional diversity and enzyme activities compared with DAP alone. This reduction in fertilizer dependency may translate into lower production costs while simultaneously decreasing the environmental impacts associated with excessive phosphorus application, including the eutrophication and depletion of finite phosphate rock reserves [168,169,170].

Numerous greenhouse and field studies have demonstrated that PSB inoculation increases plant phosphorus uptake, biomass accumulation, and crop yield under reduced phosphorus fertilization regimes [171,172]. Enhanced nutrient acquisition frequently improves fertilizer use efficiency while maintaining crop productivity, thereby increasing the economic return per unit of fertilizer applied. In addition to immediate improvements in crop performance, continued stimulation of microbial phosphorus cycling contributes to the gradual accumulation of biologically active phosphorus pools and improved soil fertility. Unlike mineral fertilizers that provide short-term nutrient availability, PSB promote long-term nutrient recycling through sustained microbial activity, potentially reducing fertilizer requirements over successive growing seasons. Consequently, the economic benefits of PSB should be evaluated not only through annual yield gains but also through improvements in soil health, nutrient sustainability, and long-term farm profitability [173,174].

Commercial interest in PSB has increased considerably during the past decade owing to growing demand for sustainable agricultural inputs and environmentally friendly nutrient management technologies. Numerous commercial biofertilizer products currently incorporate phosphate-solubilizing microorganisms either as single-strain inoculants or as multifunctional microbial consortia capable of promoting nutrient acquisition through complementary mechanisms. Among the most frequently utilized bacterial genera are *Bacillus*, *Pseudomonas*, *Paenibacillus*, *Rhizobium*, and *Enterobacter*, owing to their well-documented P-solubilizing ability, rhizosphere competence, and compatibility with diverse cropping systems [175,176].

Successful commercialization depends not only on microbial efficacy but also on formulation technology. The selection of suitable carrier materials, including peat, lignite, vermiculite, biochar, compost, and polymer-based matrices, directly influences bacterial survival, shelf life, and field performance. Liquid formulations have gained increasing attention because of their ease of application, higher cell viability, and compatibility with modern irrigation systems [177]. More recently, microencapsulation and polymer-assisted delivery systems have emerged as promising approaches for protecting bacterial cells against desiccation, ultraviolet radiation, temperature fluctuations, and other environmental stresses during storage and field application [178,179]. Furthermore, the development of microbial consortia combining PSB with nitrogen-fixing bacteria, potassium-solubilizing microorganisms, arbuscular mycorrhizal fungi, or biocontrol agents has attracted considerable interest because multifunctional inoculants may provide greater agronomic stability than single-strain products under heterogeneous field conditions [180,181].

### 5.1. Cost–Benefit Considerations and Challenges

The economic feasibility of PSB application depends on balancing inoculant production costs against improvements in crop productivity and reductions in fertilizer expenditure. Available field evidence indicates at least two economically distinct application strategies: PSB may supplement conventional fertilization to increase yield and profitability, or enable partial substitution of mineral P while maintaining acceptable productivity. These pathways should be evaluated separately because increased yield following PSB supplementation does not necessarily demonstrate fertilizer savings. The magnitude of these economic benefits varies according to crop species, soil properties, climatic conditions, fertilizer management practices, and the effectiveness of the selected bacterial strains [182,183,184].

PSB may also be co-inoculated with other functionally complementary microorganisms in deliberately assembled synthetic microbial communities (SynComs), rather than being applied exclusively as single strains. Such SynComs can integrate phosphorus solubilization with complementary plant growth-promoting functions, including biological nitrogen fixation, phytohormone production, and stress alleviation. Functional complementarity among community members may thereby enhance nutrient acquisition and plant performance under variable environmental conditions [22,28,185]. However, the agronomic and economic advantages of SynComs over single-strain inoculants remain context dependent and require further field validation.

For smallholder farmers, particularly in developing regions where phosphorus fertilizers represent a major production expense, PSB-based biofertilizers may provide a strategy for improving resource-use efficiency while potentially reducing dependence on imported mineral fertilizers [186]. However, improved P availability or crop growth alone does not establish an economic advantage. Available studies show that PSB may generate economic benefits either by improving returns when added to existing fertilizer programs or by permitting partial replacement of mineral P, and these outcomes should be distinguished when assessing commercial value (Table 2). Comprehensive economic assessments remain relatively scarce, and future research should incorporate detailed techno-economic analyses, including inoculant production and application costs, achievable fertilizer savings, yield responses and stability, net returns, benefit–cost ratios, and return on investment across different agricultural systems and growing seasons. Such analyses are essential for evaluating the practical feasibility of large-scale adoption and identifying economically optimal application strategies [187,188].

Despite substantial scientific progress, several challenges continue to limit the widespread commercialization of PSB. One of the principal constraints is the inconsistency of field performance. While many PSB strains exhibit high phosphate-solubilizing activity under laboratory conditions, their effectiveness often declines under field environments because of variations in soil physicochemical properties, native microbial competition, climatic conditions, crop genotype, and agricultural management practices [189,190]. This laboratory-to-field performance gap represents a major commercialization constraint because product efficacy ultimately requires reproducible performance across soils, crops, seasons, and management conditions rather than high in vitro P-solubilization capacity alone.

The production of commercially reliable inoculants also requires careful optimization of fermentation processes, carrier materials, formulation stability, and storage conditions to ensure adequate cell viability throughout the product shelf life. Maintaining high microbial populations during storage and transportation remains a significant technological challenge, particularly under tropical and arid environments where elevated temperatures accelerate bacterial mortality [191]. Accordingly, commercially relevant evaluation should consider viable-cell concentration throughout the declared shelf life and, where possible, retention of functional P-solubilizing activity after storage. These parameters are particularly important when translating experimental inoculum performance into commercially formulated products (Table 2).

Additional barriers include inconsistent regulatory frameworks governing microbial biofertilizers across different countries, limited quality control standards, variable product performance, and relatively low farmer awareness regarding microbial inoculants. Regulatory requirements may differ substantially among jurisdictions in permitted microbial taxa, viable-cell thresholds, functional efficacy criteria, labeling, and routes to market. For example, India specifies quantitative viability and P-solubilization requirements for PSB products, whereas the current harmonized EU framework includes only a restricted range of microorganisms, leaving many commonly investigated PSB taxa outside the harmonized route (Table 2). These factors can increase product-development and registration costs, complicate market entry, reduce confidence in biofertilizer technologies, and hinder market expansion. Furthermore, the absence of widely harmonized protocols for efficacy evaluation complicates comparisons among commercial products and limits the transferability of efficacy claims across jurisdictions and production environments. Addressing these scientific, technological, regulatory, and socioeconomic constraints will be essential for increasing farmer adoption and establishing PSB as reliable components of sustainable nutrient management systems [192,193].

**Table 2:** Commercialization, economic performance, and regulatory context of phosphate-solubilizing bacteria (PSB).

Evidence/System	Organism/Formulation	Key Quantitative Evidence	Commercial/Economic Significance	Reference
Commercial bacterial consortium: Mammoth P™	*Enterobacter cloacae*, *Citrobacter freundii*, *Pseudomonas putida*, *Comamonas testosteroni*	Greenhouse trials: wheat aboveground biomass comparable to conventional inorganic fertilization; fescue productivity increased 74–91% when Mammoth P™ or its metabolites were combined with fertilizer. Experimental inoculum ≥ 10^8^ CFU mL^−1^ vs. marketed product 2.02 × 10^6^ CFU mL^−1^; declared shelf life > 2 years.	Demonstrates translation to a marketed consortium, but the experimental–commercial inoculum difference and manufacturer involvement in the study support the need for independent field validation.	Baas et al. [194]
Liquid PSB formulation: potato	*Bacillus licheniformis* PRPSB10 + *Pseudomonas putida* PRPSB38; CMC/trehalose-protected formulations	Viability remained approximately 10^8^ CFU mL^−1^ after 365 d. Two-year field trial: yield increased ~16.1–16.2%; net return increased from ₹169,165 to ₹214,041 ha^−1^ and B:C from 2.52 to 2.92. Both compared treatments received 100% NPK + FYM.	Links shelf life with field efficacy and profitability, but PSB supplemented rather than replaced full fertilization.	Bansal, Vyas and Sharma [180]
Integrated P management: pearl millet	*Bacillus* sp. MN54 + 50% inorganic P + 50% organic P	PSB treatments produced net returns of USD 1169.7 and 1569.4 ha^−1^ and B:C ratios of 2.6 and 3.0 in 2020 and 2021; corresponding no-PSB treatments: USD996.6 and 1259.7 ha^−1^, B:C 2.4 and 2.6.	Demonstrates improved profitability of PSB within an integrated P-management strategy.	Majeed et al. [160]
PSB-assisted use of rock phosphate: maize–wheat	*Pantoea cypripedii* PSB-3 + *Pseudomonas plecoglossicida* PSB-5 with rock phosphate	Two-year trial across three agroclimatic sites; PSB + rock phosphate improved crop growth, yield, P uptake and soil P and was reported as more economical than DAP-based fertilization.	Supports a commercialization pathway in which PSB facilitate use of lower-cost rock phosphate; multilocation testing strengthens agronomic relevance.	Kaur and Reddy [86]
Global biofertilizer market	Nitrogen-fixing, phosphate-solubilizing and other microbial biofertilizers	Peer-reviewed reviews report a global market of approximately USD 1.5–2.8 billion in 2022, with projected annual growth of ~10.9–12.8%; a separate estimate projects ~USD 5.02 billion by 2030.	Indicates expansion of the broader biofertilizer sector, but these values are not PSB-specific and ultimately derive from commercial market-research sources.	Fadiji et al. [157]
India: PSB product standards	Carrier/powder and liquid PSB formulations	Product specifications: 5 × 10^7^ CFU g^−1^ for carrier/powder and 1 × 10^8^ CFU mL^−1^ for liquid formulations; P-solubilization criterion ≥30 mg P L^−1^ using tricalcium, aluminum, or iron phosphate.	Provides an explicit regulatory framework combining viability and functional P-solubilization criteria for commercial PSB products.	Malusá, and Vassilev, [194]
European Union: harmonized regulatory route	Microbial plant biostimulants under PFC 6(A)/CMC 7	CMC 7 currently lists *Azotobacter* spp., mycorrhizal fungi, *Rhizobium* spp., and *Azospirillum* spp.; common PSB genera such as *Bacillus* and *Pseudomonas* are outside the current harmonized list.	Restricts the harmonized EU route for many PSB taxa, but does not prohibit marketing through applicable national/mutual-recognition routes; expansion of CMC 7 is under evaluation.	Regulation (EU) [195]

### 5.2. Future Commercialization Perspectives

Future commercialization of PSB may increasingly benefit from advances in systems biology, microbiome engineering, and digital agriculture. Rather than relying exclusively on single bacterial isolates, next-generation biofertilizers may incorporate synthetic microbial consortia designed to perform complementary functions, including phosphorus mobilization, biological nitrogen fixation, stress tolerance, disease suppression, and phytohormone production. Integrating multi-omics technologies with ecological modeling could support the rational design of microbial communities with improved functional stability across diverse environmental conditions [193]. However, their commercial advantage over simpler formulations will require validation through reproducible field performance, adequate shelf life, economically meaningful fertilizer savings and yield responses, and compliance with applicable regulatory requirements.

Artificial intelligence and machine learning have potential applications in accelerating microbial strain discovery, predicting inoculant performance, optimizing formulation parameters, and identifying crop-specific microbial combinations using large genomic, environmental, and agronomic datasets. Combined with precision agriculture technologies, remote sensing, soil sensors, geographic information systems, and digital decision-support platforms, these approaches may enable site-specific selection and the application of PSB inoculants based on soil characteristics, climatic conditions, and crop nutritional requirements. Such data-driven management strategies could improve phosphorus-use efficiency and reduce unnecessary fertilizer inputs; however, their commercial value should ultimately be demonstrated through measurable improvements in prediction accuracy, fertilizer savings, yield stability, application costs, and net economic return [194].

In the longer term, microbiome engineering, synthetic biology, and AI-assisted biofertilizer design may expand the functionality and precision of PSB-based products. However, technological sophistication alone does not demonstrate commercial readiness. Successful commercialization will depend on reproducible agronomic performance, formulation stability and shelf life, scalable production costs, demonstrated benefit–cost performance, quality control, and regulatory readiness. Achieving this transition will require close collaboration among microbiologists, plant scientists, engineers, data scientists, industry stakeholders, and regulatory agencies to accelerate technology transfer and support the development of robust commercial products [188,189,190].

## 6. Conclusions

PSB have emerged as key components of sustainable nutrient management by improving phosphorus availability, enhancing nutrient-use efficiency, and promoting plant growth through multiple direct and indirect mechanisms. Beyond phosphorus solubilization, PSB contribute to root development, phytohormone regulation, stress tolerance, and rhizosphere interactions, highlighting their broader role within the plant microbiome.

Recent advances in genomics, transcriptomics, metabolomics, and microbial ecology have greatly expanded our understanding of PSB diversity and functional mechanisms. These approaches have revealed the complexity of plant–soil–microbe interactions and provided valuable insights for identifying highly efficient strains and developing next-generation microbial inoculants. However, inconsistent field performance, formulation challenges, limited shelf life, and the lack of standardized quality control continue to hinder large-scale commercialization. Moreover, evidence of improved phosphorus mobilization or crop growth should not be interpreted automatically as evidence of fertilizer savings or economic profitability.

Future progress may benefit from integrating systems biology, microbiome engineering, synthetic microbial consortia, artificial intelligence, and precision agriculture to improve inoculant selection and predict field performance across diverse environments. At the same time, advances in formulation technologies, carrier materials, and regulatory frameworks remain important for developing reliable and commercially viable PSB products. Their commercial readiness should ultimately be evaluated using measurable outcomes, including reductions in mineral phosphorus inputs, yield response and stability, formulation shelf life, benefit–cost performance, and regulatory compliance.

By bridging fundamental microbial ecology with agricultural biotechnology, PSB have the potential to become integral components of climate-smart and sustainable crop production. Continued collaboration among researchers, industry, policymakers, and farmers will be critical for translating scientific advances into practical solutions that improve phosphorus-use efficiency, enhance soil health, reduce fertilizer dependence, and strengthen global food security.

## 7. Future Perspectives

Future research should prioritize the integration of molecular ecology, functional genomics, multi-omics technologies, and field-based validation to establish robust mechanistic links between microbial function, phosphorus cycling, and crop productivity under diverse agricultural conditions. Advances in microbiome engineering, synthetic microbial consortia, artificial intelligence, and precision agriculture are expected to accelerate strain discovery, optimize inoculant design, and enable site-specific application strategies tailored to soil characteristics and crop requirements. Equally important will be the development of standardized evaluation protocols, improved formulation technologies, and harmonized regulatory frameworks to facilitate the translation of laboratory discoveries into reliable commercial biofertilizers. These multidisciplinary efforts will be essential for realizing the full potential of PSB as key components of sustainable and climate-resilient agricultural systems.

## Figures and Tables

**Table 1:** Roles of phosphate-solubilizing microbes in improving plant phosphorus nutrition and physiological responses.

PSB Strain/Species	Plant	P Source	Main P-Mobilizing Mechanisms	Plant Response and Experimental Conditions	Reference
*Rhizophagus irregularis*	*Trifolium repens*	Fe-bound P and Ca-P	Siderophore production and organic acid secretion	Increased shoot biomass and plant P nutrition (Rhizobox)	Zhu et al. [43]
*Pseudomonas* sp.	*Triticum aestivum*	Low-grade rock P	Enhanced phosphatase activity	Increased plant biomass and plant P nutrition (Pot experiment)	Biswas et al. [72]
*Burkholderia* sp.	*Triticum aestivum*	Ca-Mg-P	P solubilization and Cd immobilization	Increased P accumulation and plant growth (Solution culture)	Lu et al. [44]
*Enterobacter* sp.	*Solanum nigrum*	Soil P	Enhanced phosphatase activity, polyphosphate synthesis, pH reduction	Increased P accumulation and plant growth (Pot experiment)	Luo et al. [139]
*Pantoea rwandensis*, *Acinetobacter calcoaceticus*	*Cynodon dactylon*	Soil P	Phosphatase-mediated P solubilization	Plant biomass, P-uptake and chlorophyll (Pot experiment)	Jiang et al. [140]
*Enterobacter* sp.	*Vigna radiata*	Rock phosphate, Fe-P, Al-P	Phosphatase-mediated P solubilization	Plant P-uptake, Plant biomass, and P-use efficiency (Pot experiment)	Sen et al. [141]
*Bacillus*, *Pseudomonas*, *Mesorhizobium* spp.	*Hordeum vulgare* L.	Tricalcium phosphate	Phosphatase-mediated P solubilization	Plant P-uptake, plant biomass (Pot experiment)	Kouas et al. [67]
*Enterobacter asburiae*	*Zea mays* L.	Al-P, Ca-P, Fe-P	Solubilization of Al-P, Fe-P and Ca-P	Plant P-uptake, plant yield (Pot experiment)	Khuong et al. [142]
*Pediococcus pentosaceus*	*Arabidopsis thaliana*	Inorganic P phytate	Acid phosphatase-mediated dephosphorylation; organic-acid production, metal-ion chelation	Improved plant growth, enhanced phosphorus utilization (Laboratory/soil incubation study)	Yaling et al. [66]
*Pseudomonas fluorescens*	*Pisum sativum* L.	Tricalcium phosphate Ca_3_(PO_4_)_2_	Gluconic acid production; PQQ–glucose dehydrogenase (GCD) system; TCP solubilization	Improved plant growth, enhanced phosphorus utilization (Pot experiment)	Oteino et al. [41]
*Acinetobacter* sp.	*Arabidopsis thaliana*	Ca_3_(PO_4_)_2_	GCD-mediated acidification and Ca-phosphate solubilization	Increased P accumulation and seedling growth (Agar-plate/controlled seedling co-cultivation)	Xie et al. [52]
*Bacillus velezensis*	*Zea mays* L.	Organic P, sodium phytate	Phytase-mediated phytate degradation, phosphatase activity	Plant growth and plant P-uptake (Pot experiment)	Zhou et al. [100]

## Data Availability

No new data were created or analyzed in this study. Data sharing is not applicable to this article.
